# Patient performance assessment methods for upper extremity rehabilitation in assist-as-needed therapy strategies: a comprehensive review

**DOI:** 10.1007/s11517-025-03315-z

**Published:** 2025-02-07

**Authors:** Erkan Ödemiş, Cabbar Veysel Baysal, Mustafa İnci

**Affiliations:** 1 Department of Biomedical Engineering, Çukurova University, 01330 Sarıçam Adana, Turkey; 2 Department of Mechatronics Engineering, İskenderun Technical University, 31200 Hatay, Turkey

**Keywords:** Robotic rehabilitation, Upper extremely rehabilitation, Assist-as-needed, Performance assessment, A comprehensive review

## Abstract

This paper aims to comprehensively review patient performance assessment (PPA) methods used in assist-as-needed (AAN) robotic therapy for upper extremity rehabilitation. AAN strategies adjust robotic assistance according to the patient’s performance, aiming to enhance engagement and recovery in individuals with motor impairments. This review categorizes the implemented PPA methods in the literature for the first time in such a wide scope and suggests future research directions to improve adaptive and personalized therapy. At first, the studies are examined to evaluate PPA methods, which are subsequently categorized according to their underlying implementation strategies: position error–based methods, force-based methods, electromyography (EMG), electroencephalography (EEG)-based methods, performance indicator–based methods, and physiological signal–based methods. The advantages and limitations of each method are discussed. In addition to the classification of PPA methods, the current study also examines clinically tested AAN strategies applied in upper extremity rehabilitation and their clinical outcomes. Clinical findings from these trials demonstrate the potential of AAN strategies in improving motor function and patient engagement. Nevertheless, more extensive clinical testing is necessary to establish the long-term benefits of these strategies over conventional therapies. Ultimately, this review aims to guide future developments in the field of robotic rehabilitation, providing researchers with insights into optimizing AAN strategies for enhanced patient outcomes.

**Graphical Abstract:**

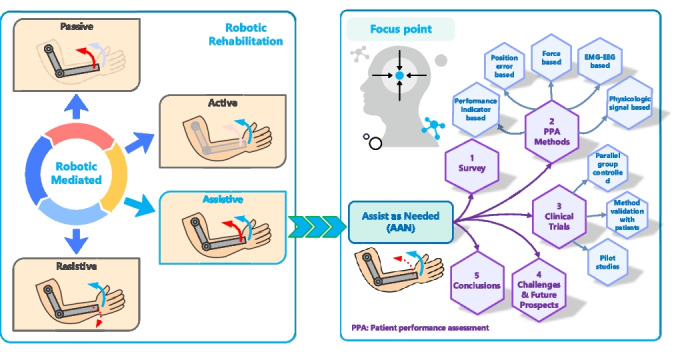

## Introduction

Damage to the central nervous system caused by aging or disease in individuals leads to motor dysfunction and adversely impacts their activities of daily living (ADL). Patients with motor impairment require intensive, repetitive, and personalized rehabilitation therapies to restore all or part of the motor functions [1]. A common method used in treating patients who have motor dysfunctions is conventional therapy, where exercises are performed with physical therapists. However, this method cannot always provide sufficient treatment due to the need to coordinate multiple therapists and the increased workload resulting from a large patient population. To overcome these limitations, performing exercises with robotic devices has emerged as a new method to provide patients with intensive, repetitive, and person-adapted therapy exercises [2, 3]. The effectiveness of robotic rehabilitation in treating patients with motor dysfunction has also been demonstrated in clinical trials [4, 5].

**Fig. 1:**
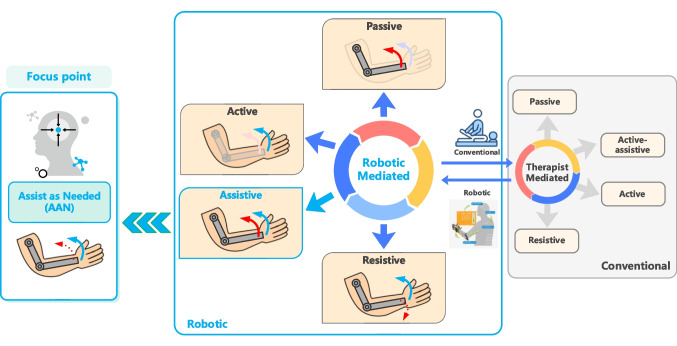
The main therapy strategies and implementations of robotic rehabilitation

Robotic rehabilitation devices can be grouped according to the part of the body in which they are implemented or their mechanical structure [6, 7]. Based on rehabilitation mode, robotic therapeutic devices can be divided into four groups: passive, active, resistive, and assistive [8]. In passive rehabilitation mode, the exercise is performed by the robotic device, requiring no effort from the patient. In active mode, the patient performs the exercises without any assistance from the robotic device. The robotic rehabilitation device applies contrary or disruptive forces to the patient’s movements in the resistive mode, making the therapy exercises more challenging for the patients. In assistive exercises, the patient performs the desired therapy tasks with the assistance of a robotic device. The active and resistive rehabilitation modes may be suitable for capable patients; however, they might not be appropriate for those who cannot complete the exercises by themselves or exert the required effort. Also, clinical studies reveal that passive exercises have limited benefits for patients with motor impairments [9, 10]. Additionally, passive exercises restrict patients’ active participation, which is a crucial factor in enhancing functional outputs gained from therapy [11]. In the assistive rehabilitation mode, the assist-as-needed (AAN) approach has emerged to make exercises both feasible and beneficial for patients with varying motor capabilities, as well as to increase patient active participation [12], as shown in Fig. 1.

In the AAN strategy, the robotic assistance provided to the patient is determined based on patient performance, to improve the patient’s engagement. Additionally, therapy tasks and their difficulty levels are adjusted based on the patient’s performance to ensure the tasks are sufficiently challenging for the patient. In this strategy, robotic assistance is applied when the patient deviates from the desired therapy trajectory or cannot complete the rehabilitation exercise. This provides the patient with as much timely assistance as necessary, enabling them to participate more actively in therapy exercises and aiming to prevent passive training therapies that do not produce much functional output.

The core of AAN strategies lies in the patient performance assessment method, which is essential for determining the level of robotic assistance provided to the patient and adjusting therapy task difficulty. In the existing literature, numerous review studies were performed related to robotic rehabilitation devices [13–17]. Proietti et al. [18] have examined the control strategies implemented in upper limb rehabilitation devices. Miao et al. [19] analyzed high-level control techniques used in upper limb robotic training. In another study, lower limb robotic devices were examined and compared in terms of rigid exoskeletons and soft robotic devices [20]. In another review study conducted by Maciejasz et al. [21], robotic rehabilitation devices were discussed in terms of their mechanical designs, control strategies, and clinical applications. Also, Güçlü and Çora examined hand rehabilitation devices based on their mechanical designs, actuator types, and control strategies [22]. However, to the best of our knowledge, none of the previous research has examined the patient performance evaluation methods implemented in the AAN strategies.

In this study, the existing patient performance assessment (PPA) methods used in AAN strategies applied in the increasingly growing field of upper extremity robotic rehabilitation in recent years [23] are explored to present the implemented approaches for performance evaluation and determining robotic assistance. Contrary to the existing review papers, the current study presents the classification of PPA methods with advantages and limitations. Additionally, this study reviews and classifies methods that were tested with patients based on their clinical outcomes and implementation approaches, and also provides detailed guidance to the researchers for future studies. In this review study, more than 100 scientific studies published up to 2024 have been analyzed using major databases such as WoS, IEEE, ScienceDirect, Springer, Wiley, Taylor&Francis, and Google Scholar. The research focused on the assistive therapy approach, specifically on AAN strategies implemented on the upper extremity robotic devices, and a total of 51 studies analyzed have been included in this review. The inclusion criteria for the study were selected as the focus on upper extremity rehabilitation, the incorporation of AAN strategies, and a clear discussion of the implemented PPA methods. To summarize, the major contributions of this paper are given as follows:

- This review provides a comprehensive analysis of the PPA methods used in AAN strategies applied to upper extremity robotic rehabilitation devices, addressing the advantages and limitations of existing methods in the literature with such broad coverage for the first time.
- Clinically tested AAN strategies are classified, and their clinical outcomes and implementation approaches are presented in detail.
- By proposing suggestions for the limitations of existing performance evaluation methods, this review offers recommendations and new research avenues that will guide future studies in the field.

The remainder of this paper is laid out as follows: In “Section 2,” the existing PPA methods are classified and analyzed. “Section 3” presents the clinically tested methods along with their clinical outcomes. Challenges and future prospects are provided in “Section 4.” Finally, the conclusions are given in “Section 5.”

## Patient Performance Assessment (PPA) methods: classification

**Fig. 2:**
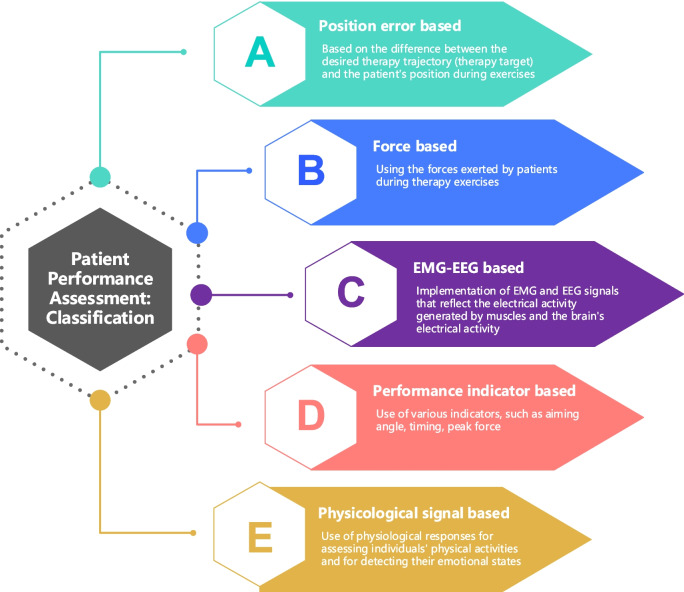
Classification of PPA methods implemented in AAN strategies

This section proposes a categorization of the existing PPA methods implementing AAN strategies. The performance evaluation methods of upper extremity rehabilitation devices are handled and classified into five groups based on their implementation approaches: position error–based methods, force-based methods, EMG- and EEG-based methods, methods using performance indicators, and physiological signal–based methods, as shown in Fig. 2.

### Position error–based PPA methods

Position error–based methods mainly evaluate patient performance based on the difference between the desired therapy trajectory (therapy target) and the patient’s position during exercises. The error signal is a clear metric and has simple computational complexity. Also, error is a key signal for human motor relearning during rehabilitation exercises [24]. Therefore, it is the most widely used performance evaluation method in AAN approaches in the existing studies. However, relying exclusively on tracking error signals to evaluate a patient’s capabilities or therapy performance can lead to misleading results, especially when the error signal is minimal due to robotic assistance, even though the patient lacks motor skills. Also, this misjudgment may impact the patient’s active participation, potentially leading to slacking behavior.

**Fig. 3:**
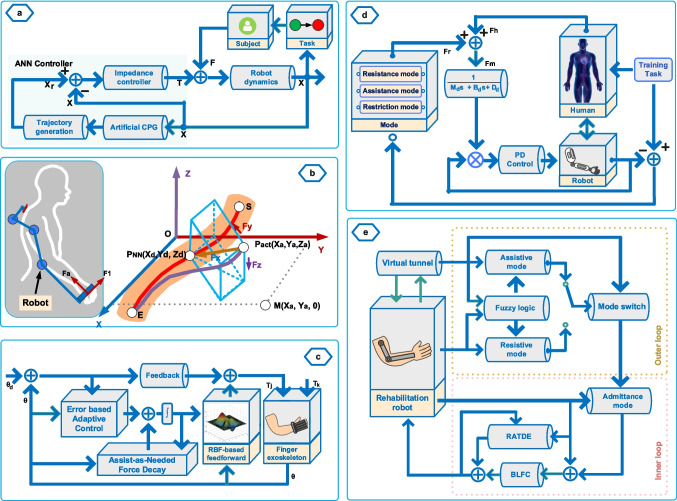
Some implementations of position error–based PPA methods

Position error–based performance evaluation methods include several application approaches: simple position error–based, advanced position error–based, virtual tunnel–based, and therapy mode–implemented position error–based methods. Additionally, the implementation schemes of some methods are provided in Fig. 3. In simple position error–based methods, patient performance is evaluated solely on the position error signal. As a simple position error–based method, Asl et al. [25] presented a strategy that evaluates patient performance based on tracking errors using two approaches: with and without a force sensor. In another approach developed for hand rehabilitation, Bower et al. [26] proposed a method for patients with varying levels of impairment across different movements. In this study, using radial basis functions (RBF), the patients’ state-dependent (position and direction) impairments of abilities were assessed based on error signal, and the assistance to be provided to the patient was determined accordingly. In another study conducted by Peng et al. [27], while performance was evaluated based on position error, the estimation of patient movement intention was also included in the developed method to enhance participation in therapy exercises. In this approach, the frequency and phase of the patient’s movement were estimated in real time using artificial central pattern generation (CPG), as shown in Fig. 3a. Additionally, the authors further developed their method by proposing another approach in which patient movement patterns are estimated using an adaptive frequency oscillator (AFO) as a sensorless estimator. In this approach, the error is used to determine the assistance provided to the patient, and therapy tasks are adjusted according to patient performance [28].

In advanced position error–based methods, additional factors are incorporated into the performance evaluation to prevent potential drawbacks from solely assessing performance based on the error signal. Guo et al. [29] proposed a method in which patient performance is evaluated using the trajectory tracking error signal, and patient assistance was provided through position-based and velocity-based impedance controllers. Additionally, to overcome the potential issues arising from evaluating patient performance based only on the error signals, a task performance function based on position tracking error and assistive force has been defined, which is used to evaluate the patient’s motor capacity and determine the desired trajectory velocity. In another study, Widanage et al. [30] contributed to using position error for therapy performance assessment by introducing a new approach developed for a teleoperated rehabilitation robotic system, in which patient performance and the assistance force provided to the patient were determined based on position error, velocity, and vibration constraints. Also, Zarrin et al. [31] proposed a different method in which patient performance is evaluated using error signals and interaction forces.

Establishing a virtual force or velocity tunnel around the therapy trajectory is one of the methods applied in robotic rehabilitation strategies [18]. This approach aims to provide the patient with spatial freedom during exercises or to prevent significant position and velocity errors. One of the virtual tunnel approaches applied in AAN methods that evaluate patient performance based on position error was realized by Zhang et al. [32], by implementing a virtual tunnel that allows the patient freedom of movement around the desired therapy path, as illustrated in Fig. 3b. In this method, the assistive force was applied when the patient deviated outside the virtual tunnel. Agarwal and Deshpande developed and compared two methods to fulfil different therapy needs, learned force-field control and adaptive assist-as-needed control, for a hand rehabilitation device named Maestro [33]. In the learned force field control, joint torques were calculated with a neural network using joint angles, and then a force field was created around the desired trajectory to assist the user based on the deviation from the target path. In adaptive assist-as-needed control, the assistance force provided to the patient was determined using an RBF network based on the trajectory tracking error signal, as given in Fig. 3c. The experimental findings indicated that the adaptive assist-as-needed controller approach was more suitable for tasks where timing and desired trajectory tracking are critical. Keller et al. [34] presented another example of error-dependent performance evaluation. In this study, impedance controller stiffness was adjusted using gain scheduling to ensure that the patient approached the target position at a predefined level of precision, and direction-dependent supportive force was used to assist the patient along the desired trajectory. This method also includes a virtual tunnel to keep the patient close to the desired trajectory path and minimum and maximum speed restrictions to prevent the user movement from being too slow or fast.

Patients with different levels of motor impairment may require other therapy strategies such as passive or resistive. To meet this need, some position error–based performance evaluation methods have implemented different therapy modes in addition to the AAN approach. Lin et al. [35] developed an AAN strategy that can implement active, passive, and resistive rehabilitation modes alongside the assist-as-needed approach by adjusting controller parameters for wrist rehabilitation. In this method, patient performance was determined based on tracking errors of wrist flexion/extension and radial/ulnar joints, and the assistive force was adjusted through the stiffness and damping parameters of the impedance controller. Zhang et al. [36] proposed a method similar to active, passive, and AAN therapy modes, which can perform three different modes: the zero interaction force (ZIF) mode, the restriction interaction region (RIR) mode, and the AAN mode. In this method, switching between therapy modes and evaluating patient performance was realized using the trajectory tracking error signal. In another approach, Li et al. [37] presented a structure that includes resistance, assistance, and restriction therapy modes, as given in Fig. 3d. In this approach, therapy mode transitions and patient performance evaluations were performed based on a fuzzy logic system that takes error and error rate as inputs. Also, Guo et al. [38] have introduced a method incorporating assistive and resistive therapy modes to increase the participation of patients with partial strength and control abilities in treatment using the resistive mode. In this approach, transitions between assistive and resistive modes, and the assistance or challenging effects provided to the patient during these modes, were determined based on the patient’s performance. A fuzzy logic system was implemented to evaluate patient performance, based on mean task path tracking error and mean velocity data, as shown in Fig. 3e.

### Force-based PPA methods

**Fig. 4:**
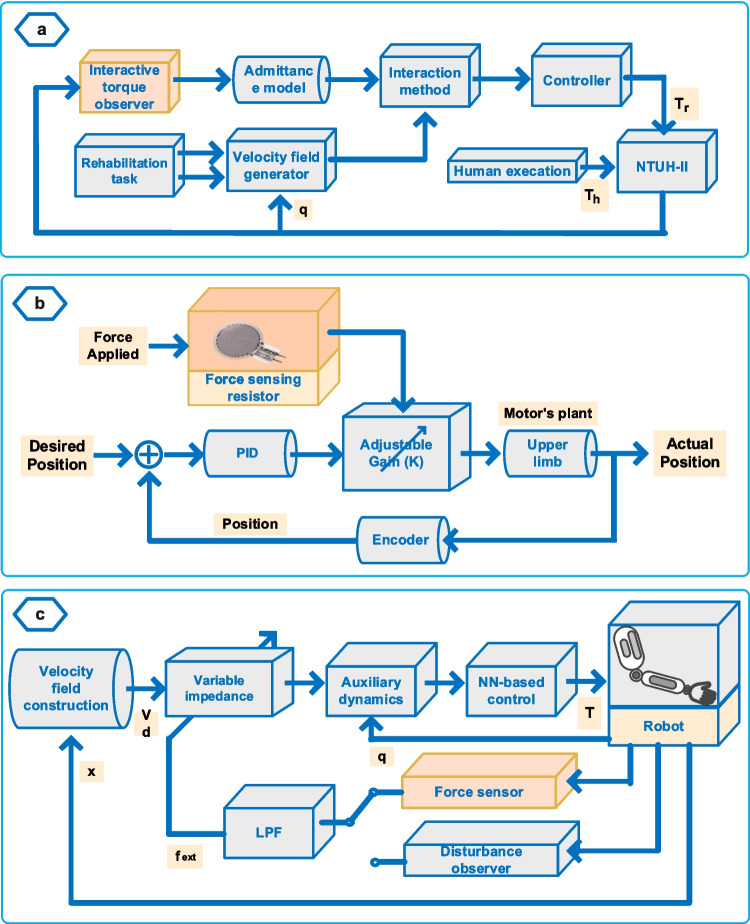
Examples of force estimation and measurement-based PPA methods

The forces exerted by patients during therapy exercises represent the muscular capabilities of the patients, and using muscle force is one of the applied methods to evaluate patient performance during assistive therapy exercises performed with robotic devices. Patient-exerted forces can be measured directly during the exercises or inferred through patient-robot interaction forces. The force-based PPA methods used in the AAN strategy can be divided into two groups: force estimation approaches and force measurement approaches. Application examples for both approaches are illustrated in Fig. 4.

The force estimation approach is based on predicting the patient’s force using various techniques to assess patient capability. A major limitation of this approach is that patient capability estimation relies on the dynamic model of the robotic devices. As the mechanical structure of the robotic device becomes more complex, accurately assessing patient performance becomes increasingly difficult. Also, this method’s reliance on device design and dynamics makes it device-specific and poses challenges for its application with other therapy devices. Pehlivan et al. [39] propose a minimal-assist-as-needed (mAAN) method where patient inputs are evaluated sensorless through rehabilitation device dynamics using Kalman filter and Lyapunov stability analysis. Additionally, this method aims to increase the patients’ active participation with the allowable error-bound modification algorithm and the decayed disturbance rejection algorithm used to prevent the sensorless force estimation method from restricting capable patients. Chia et al. [40] developed another method for sensorless force estimation to evaluate patient performance. In this method, a Kalman filter–based interactive torque observer is used to estimate the patient's interactive torque, as given in Fig. 4a. Subsequently, this torque value is converted into the patient’s active velocity using an admittance model. The patient’s active velocity is evaluated with the assistive velocity, obtained from the velocity created around the desired therapy trajectory, to calculate the time-decoupled assistance to be provided to the patient.

On the other hand, one method for obtaining patient force is through the use of a force/torque sensor. However, using sensors has disadvantages such as high sensor costs or limitations in sensor placement that can restrict patient-robot interaction. In one of the studies evaluating patient performance using sensors, Azman and Lukman proposed a simple and easy-to-implement AAN strategy to assist the patient with basic movements in the eating activity [41]. In this method, as given in Fig. 4b, the patient level of capability (LOC) was determined according to the force exerted by the patient during exercise measured by the force sensing resistor, and the level of assistance was adjusted by a proportional-integral-derivative (PID) controller gain parameter. In another study, an AAN method was proposed that ensures task completion when patients cannot complete exercises due to motor skill deficiencies [42]. In this strategy, two different methods were applied: force sensor–based and disturbance observer–based methods, as shown in Fig. 4c. In the force sensor–based method, patient inputs were predicted using force sensor information, and this method does not require knowledge of human–robot system dynamics. In the disturbance observer–based method, knowledge of robot dynamics was required, and a neural network (NN) was used to compensate for the effects imposed by the human subject on the system.

### EMG-EEG-based PPA methods

**Fig. 5:**
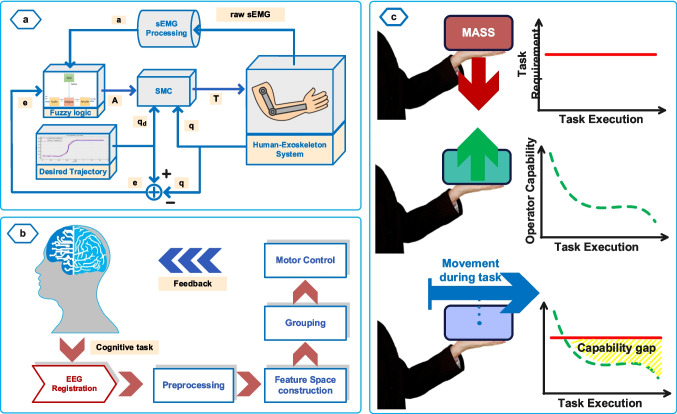
EMG and EEG signals implementations for PPA

The electromyogram (EMG) signals reflect the electrical activity generated by muscles and are biological signals produced as a result of motor commands sent to muscle motor units during muscle contraction [43]. EMG signals can be measured using transcutaneous electrodes placed on the skin surface or percutaneous intramuscular electrodes through invasive methods from within the muscle. In AAN strategies, EMG signals are used to evaluate patient performance by calculating muscular capability such as the force exerted by the patient, or determining the motion intention [44, 45]. Additionally, electroencephalography (EEG) signals, which represent the brain’s electrical activity and are measured using electrodes placed on the scalp, are used to determine patient motion intention. Some implemented methods that use EMG and EEG signals for patient performance evaluation are also illustrated in Fig. 5. Due to their higher signal-to-noise ratios and increased robustness, particularly during movement, EMG signals are more frequently preferred over EEG signals in movement intention assessment applications [46]. However, there are challenges in measuring EMG and EEG signals, such as low signal-to-noise ratios, sensitivity to skin conditions like sweating, hair, and fat layers, as well as being affected by movement artifacts [47, 48]. Additionally, the small variations in EMG signals that occur during fine movements, such as finger and wrist motions, make it challenging to utilize this signal effectively for exercises targeting these regions [46].

In an EMG-EEG-based type, Teramae et al. [49] conducted a study on using EMG signals for evaluating patients’ performance during therapy exercises. In this study, the patient’s joint torques were calculated using surface EMG signals, and the deficient joint torque required for the desired movement was provided to the patient through the AAN controller. In another study using EMG signals to evaluate patient performance, Delgado et al. [50] proposed the fuzzy sliding mode control method. In this approach, as illustrated in Fig. 5a, patients’ surface EMG (sEMG) signals and position error signals during therapy exercises were evaluated using a fuzzy inference system (FIS) and, based on the fuzzy output, assistance to be provided to the patient was adjusted. Sun et al. [51] proposed a method that utilizes the AAN approach for soft robotic gloves by measuring the patient’s sEMG signals. In this method, the patient’s muscle force for grasping movement was estimated using the radial basis function neural network (RBFNN) based on the EMG signals measured from the forearm. The assistance force to be provided to the patient was specified by subtracting the predicted patient force from the reference grasping force. The reference grasping force was determined through measurements with a healthy subject.

As an implementation of EEG signals, Kapsalyamov et al. [52] proposed an approach for implementing the AAN strategy by incorporating a brain-computer interface, as given in Fig. 5b. In this method, the subject’s total of 12 movement intentions (six directions such as up and down, and six rotations such as forward and backward) were estimated using EEG signals measured from 16 channels. To realize the AAN strategy, the interaction force between the human limb and the robotic device is aimed to be zero in the developed controller. In this approach, if the patient can move their arm, the controller aims to maintain zero interaction force. If the patient cannot move their arm, the system attempts to understand the patient’s movement intention using EEG signals. In another study conducted by Gou et al. [53], the assistance provided to the affected limb was determined based on the user’s healthy limb. In this study, the endpoint stiffness of the healthy limb was estimated using sEMG signals and human–robot interaction forces. Then, using the same approach, a stiffness model of the affected limb was created and the stiffness of the healthy limb was used as a benchmark to provide personalized assistance to the user during exercises by the AAN strategy.

As an example of using sEMG signals for fatigue assessment, Di Luzio et al. proposed an AAN strategy in which human is included in the control loop through a bio-cooperative approach [54]. In this method, patient performance was calculated as a combination of two different assessments. During the exercise, muscle fatigue was assessed using sEMG signals and a spectral fatigue index, and the arm-weight support provided to the patient was adjusted based on the level of fatigue. Additionally, the patient’s biomechanical indicators were calculated using magneto-inertial unit (M-IMU) sensors, and the assistive force was determined according to the biomechanical indicators by adjusting the controller stiffness.

Another method used to evaluate patients’ muscular capabilities is assessing patient capacity using a musculoskeletal model, without using EMG signals. As an example, Carmichael and Liu have presented an approach that utilizes a musculoskeletal model to evaluate patient performance during exercises and determine the assistance to be provided to the patient [55, 56]. In this method, the strength required to complete the therapy task was calculated using a task model. Patient capabilities were assessed with a musculoskeletal model and the difference between the force required to complete the task and the patient’s capacity was provided to the patient according to the AAN strategy, as given in Fig. 5c. The main disadvantage of this approach is the musculoskeletal model parameters were not adjusted specifically for the individual patient.

### Performance indicator–based PPA methods

**Fig. 6:**
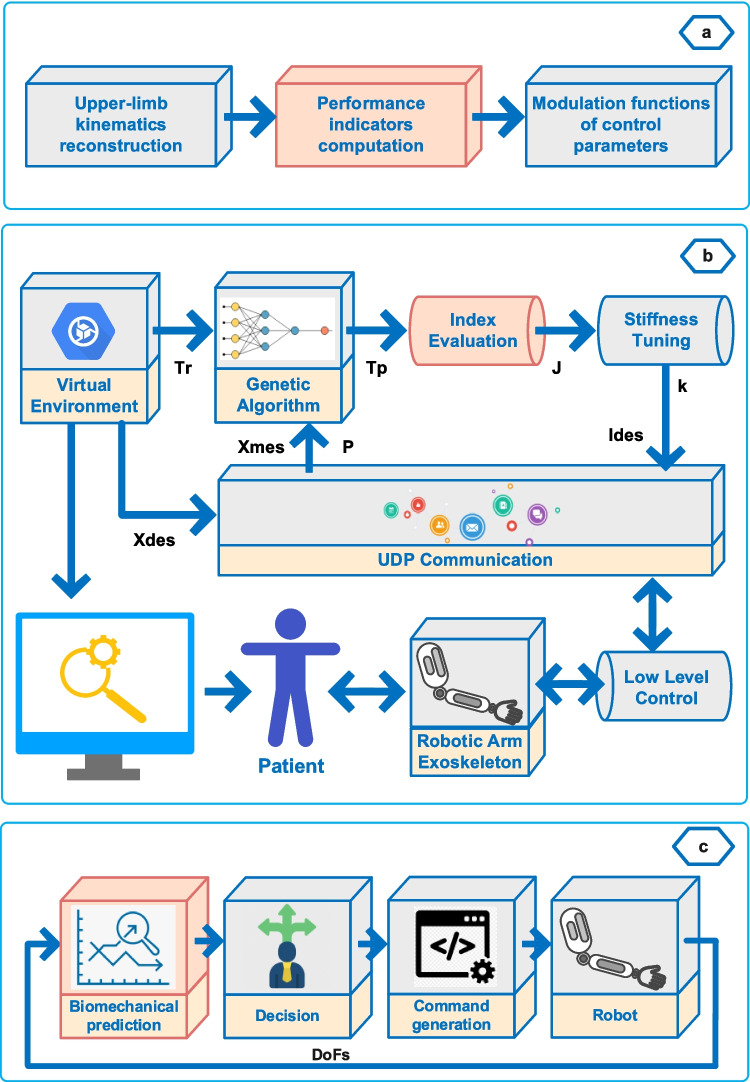
Performance indicator–based PPA methods

The performance of patients with motor dysfunctions during the therapy exercises can also be evaluated using various indicators, such as aiming angle, timing, peak force, and certain ability indices implementations, in addition to position error, force, or EMG and EEG signals. In AAN strategies, these indicators are another commonly used method for assessing patient therapy performance [57, 58]. Figure 6 presents some performance indicator–based PPA methods used in the existing studies. However, performance indicators may require adjustment for different exercises or not be suitable for various exercise types, which limits the applicability of this method across different types of therapy tasks. Papaleo et al. [59] proposed a patient-tailored adaptive strategy using biomechanical indicators such as aiming angle, useful mean force, and useful peak force to assess a patient’s therapy performance, as expressed in Fig. 6a. These indicators are computed via encoders, magneto-inertial sensors, and accelerometers. As illustrated in Fig. 6b, Stroppa et al. [58] propose a method in which the patient’s therapy performance during rehabilitation exercises was evaluated using indicators. In this method, the patient’s performance was evaluated with three different indicators calculated using a genetic algorithm, and the method was applied on an elliptical trajectory.

Another example of using indices in determining patient performance is implemented by Mounis et al. through the progress-based assist-as-needed (pAAN) approach [60]. In this study, a functional ability index (FAI) is presented, adapted from the Wolf Motor Function Test, which determines patient performance based on four indices: speed, time to complete a desired task, the amount of deviation from the trajectory, and the amount of effort (physical attempt). In a simulation study conducted by Pérez-Rodríguez et al. [57], an anticipatory assist-as-needed algorithm is proposed. In this study, patients first performed ADL exercises without assistance establishing their biomechanical profiles using a multilayer perceptron (MLP)-based approach, as shown in Fig. 6c. Subsequently, the patients’ profiles have been compared with data from healthy individuals performing ADL exercises obtained from the literature, and using a fuzzy Bayesian decision system, the assistive force has been provided using the patient ability model before the deviation from the reference trajectory occurred.

### Physiological signal–based PPA methods

Physiological signals are important indicators both for assessing individuals’ physical activities and for detecting their emotional states [61, 62]. In the existing literature, some methods implemented the use of physiological signals to detect the emotional state or stress level of the individuals [63–66]. Also, in the field of robotic rehabilitation, studies exist where physiological responses are used to detect patients’ emotional states, and therapy exercise difficulty is adjusted based on patients’ emotional status such as arousal (represents mental alertness and physical activity), valance (also sometimes called pleasure) [61], and dominance in assistive robotic rehabilitation strategies [67, 68].

Physiological signals can also be used to evaluate patients’ therapy performance during robotic rehabilitation exercises. Since physiological signals are an important indicator for evaluating patients’ physical activity, Ödemiş and Baysal proposed a method that assesses patients’ exercise participation and performance using their physiological responses and desired trajectory tracking error signals [69]. In this method, a FIS was used to evaluate exercise performance, as well as tiredness and slacking, by using the patient’s heart rate (HR) and galvanic skin response (GSR) signals measured during the exercise along with the error signal. Because the physiological signals can vary from person to person, the method focused on the changes in physiological signals for performance evaluation, while the signal levels are examined for tiredness and slacking assessments. Nevertheless, the susceptibility of physiological responses to environmental conditions and the patient’s emotions may complicate their use in assessments. Especially, the GSR signal is sensitive to external factors such as temperature and humidity, which can introduce noise and lead to signal variability [70].

## Evaluation of PPA methods based on clinically implementation

In robotic rehabilitation, testing the developed method through clinical trials is crucial for evaluating treatment efficacy, as well as validating safety and patient outcomes [71, 72]. However, integrating robotic rehabilitation devices into clinical settings presents several challenges, such as customizing the devices to meet patient needs, adapting them to the treatment process, securing the necessary approvals, and ensuring seamless integration into the clinical environment [73, 74]. Therefore, the effectiveness of many proposed methods has not been clinically evaluated [15, 75].

In this section, 15 AAN methods proposed for upper extremity rehabilitation, which have been clinically tested for effectiveness through trials, are reviewed. Clinically applied methods are categorized into three groups: parallel group–controlled trials, method validation with patients, and pilot studies. Studies comparing the outcomes of conventional therapy and the developed method by applying them to two patient groups were categorized as parallel group–controlled trials. In studies without a control group getting traditional treatment methods, those involving more than five patients were classified as method validation with patients, while those performing with fewer than five patients were evaluated as pilot studies.

### Parallel group–controlled clinical trials

Parallel group–controlled trials are studies in which patients are divided into two groups, one receiving conventional therapy methods and the other receiving robot-mediated rehabilitation, and the outcomes from both groups are evaluated and compared using various clinical scales and measurements [73]. Parallel group–controlled trials are examined under this subsection. Studies in this group are summarized in Table 1, which also specifies the evaluation methods used to compare the findings obtained from conventional and robotic therapy methods. As an example of a parallel group–controlled trial, Chen et al. [76] proposed a structure that can perform active, passive, and assistive rehabilitation modes and also includes the AAN strategy. In this approach, the robot-to-human joint-mapping block is used to convert robot joint angles into patient joint angles, and a dynamic arm model and real-time torque measurements from the subject are implemented, allowing for the evaluation of the patient’s performance during exercises. This method has been tested in clinical trials with six stroke patients using a robotic exoskeleton with 7 degrees of freedom (DOF). During the clinical trials, one of the patients was selected as the control group, and the effectiveness of the device’s therapy modes was evaluated using the stroke rehabilitation assessment of movement (STREAM) scale. Among the five patients who performed exercises using the developed robotic system, no differences were observed in the STREAM scores of three patients. Notably, two of these patients already had STREAM scores close to the maximum value prior to the exercises. In another clinical trial, Frullo et al. [77] proposed a method that uses an RBF to predict patient force for performance assessment. The effectiveness of the method was tested in a parallel group–controlled clinical trial in elbow and wrist rehabilitation exercises using a 4 DOF robotic exoskeleton. Clinical trials were conducted with 14 incomplete Spinal cord injury (SCI) patients, divided into two groups: the AAN group, where patients performed exercises using the developed system, and the subject-triggered (ST) group, where a therapist determined therapy difficulty and assistive force. After ten sessions of therapy exercises, no significant difference was found between the groups in the action research arm test (ARAT) results. While the quality of movement improved in both groups, the AAN group demonstrated greater enhancement in robotic-assessed joint movement quality compared to the ST group.

**Table 1:** AAN strategies tested through parallel group–controlled clinical trials

Study	Clinical trial group	PPA method	#Participants	Robotic device	Assessment method
Chen et al. [76]	Parallel group–controlled trials	Force measurement	6 – 1 as CG^*^	NTUH-ARM 7DOF	STREAM scale
Frullo et al. [77]	Parallel group–controlled trials	Force estimation	14 – 7 as CG^*^	MAHI EXO-II 4 DOF	ARAT test
Peng et al [78]	Parallel group–controlled trials	Position error/virtual tunnel–based	24– 12 as CG^*^	CASIA-ARM	Fugl-Meyer assessment scale
Wolbrecht et al. [79]	Parallel group–controlled trials	Position error–based	26 – 13 as CG^*^	PNEU-WREX 4DOF	Fugl-Meyer score, grip strength

^*^CG control group

Peng et al. [78] introduced a method that evaluates patient performance based on the trajectory error signal and tested the method’s effectiveness through clinical trials. In this approach, a virtual force tunnel was used to allow the patient freedom of movement around the trajectory, while an assistive force that varies based on the user’s error signal was provided. In this method, the developed controller is also capable of implementing an active resistive therapy mode in addition to the active assistive mode. The effectiveness of this method was tested through clinical trials with 24 patients who had motor impairments after a stroke, with 12 patients in the experimental group and 12 in the control group. During the clinical trials, both groups received routine therapy, with an additional robotic therapy provided to the experimental group. The robotic therapy involved at least 20 min of training, five times a week for 4 weeks, totaling 20 sessions using an end-effector-based robotic rehabilitation device. The control group received an equal duration of conventional training. To assess the patients’ progress during the experiments, the upper extremity section of the Fugl-Meyer assessment (FMA-UE) scale was used. The clinical findings showed that patients in both groups had significant gains in FMA-UE scores, with higher changes seen in patients assigned to robotic therapy compared to those in conventional therapy. However, there were no significant differences between the two groups in terms of the outcomes. In another study, Wolbrecht et al. [12] proposed an assist-as-needed approach in which the patient’s abilities were learned using RBF based on the tracking performance. In this method, the assistive force was adjusted based on the patient’s trajectory-tracking performance. In this approach, the forgetting factor, which decays the assistive force when the patient can complete the movement without assistive force or the error signal is small, was also included in the developed controller, aiming to prevent the patient from slacking and increase active participation. The method was tested with 11 chronic stroke patients, and also a parallel group–randomized controlled clinical trial [79]. The 26 chronic stroke patients who participated in clinical experiments were divided into two groups, the robot group and the control group, and 24 training sessions were conducted with the patients approximately three times a week for 8 to 9 weeks. The robot group received robot-assisted training using the Pneu-Wrex device, while the control group participated in conventional therapy exercises under the supervision of a physical therapist. The results of the study showed greater improvements in measures such as Fugl-Meyer score, grip strength, and box and blocks score in robot-assisted training compared to traditional therapy.

### Method validation with patients

Another approach used in clinical studies is evaluating the effectiveness of the method based on patient outcomes at the end of the therapy process, without using a control group. In this section, clinical studies that evaluated the effectiveness of the method through experiments conducted with five or more patients, but without a control group, are reviewed. These researches are also outlined in Table 2. Luo et al. [80] have presented a greedy AAN (gAAN) scheme using RBF networks to model patients’ functional capacities. The RBF network modeled the patient’s motor capacity based on maximum forces during rehabilitation exercises, and to increase active participation and prevent slacking, the weight vectors of the RBF network were updated according to the patient’s performance. In this method, the assistance provided to the patient was adjusted based on the patient’s capacity evaluated by the RBF network and the trajectory tracking error signal during exercises. The gAAN scheme was tested with clinical trials involving 12 subjects with neurological impairments using a 2 DOF planar rehabilitation robot, and the results indicated that the RBF network encourages active participation of patients. In another study, the functional activity spline function (FASF) technique was introduced to determine patients’ movement abilities and adjust robotic assistance based on patients’ skills [81]. The FASF algorithm used parameters such as exercise completion time and quality of movement to determine patients’ functional ability. To demonstrate the method’s effectiveness, clinical experiments were carried out with 15 hemiplegic patients on two different tasks (pick-and-place and table-to-mouth tasks) using a 2-DOF upper limb rehabilitation exoskeleton. The developed method successfully assessed the capacity of patients per clinical measurements. Additionally, the level of assistive torque provided to patients was adjusted according to their impairment level, based on their estimated functional ability.

**Table 2:** Clinical trials performed through method validation with patients

Study	Clinical trial group	PPA method	#Patients	Robotic device
Luo et al. [80]	Method validation with patients	Position error–based	12	CASIA-Arm 2 DOF planar robot
Mounis et al. [81]	Method validation with patients	Performance indicator–based	15	2 DOF rehabilitation exoskeleton
Tamantini et al. [82]	Method validation with patients	Physiological signal–based	8	KUKA light weight robot
Ödemiş and Baysal [83]	Method validation with patients	Physiological signal–based	10	None

Tamantini et al. [82] realized one of the studies using physiological responses in the AAN approach. In this method, the controller stiffness used to adjust the assistance provided to the patient was determined based on the patient’s kinematic performance and psychophysiological state assessed through physiological signals. The kinematic performance assessment was evaluated using the aiming angle, representing the difference between the patient’s position and the assigned trajectory during the exercise. The psychophysiological states regarding arousal and valence were assessed using a two-layer fuzzy logic model that incorporated the patient’s HR, GSR, and respiratory rate (RR) signals. To validate the proposed method, 20-day robot-aided rehabilitation sessions were performed with eight orthopedic patients using the KUKA end-effector-based rehabilitation device. The findings demonstrated that the psychophysiological-aware strategy was effective in providing personalized treatment and improving motor performance. Additionally, the psychophysiological adaptation factor positively impacted the patients’ interaction experience and clinical outcomes. In another clinical trial, Ödemiş and Baysal evaluated the effectiveness of their physiological signals–based method [69] through experiments conducted with ten patients suffering from frozen shoulder syndrome [83]. During the 4-week clinical trials, patients performed exercises once a week using the developed participation and performance evaluation system, in addition to the treatment protocols recommended by the doctor. Also, the patients’ progress throughout the treatment process was monitored with weekly clinical assessments, including the visual analog scale (VAS) pain scale and range of motion (ROM) measurements. The findings of the clinical trials were statistically analyzed, and it was found that the weekly performance evaluation data of the participation assessment system was consistent with the improvement of the patients during the treatment process.

### Pilot studies

Due to the challenges of clinical trials, some methods have been tested with a smaller number of patients [84–86]. Clinical studies conducted with five or fewer patients have been considered pilot studies and are additionally summarized in Table 3. In an example of pilot clinical trials, the FAI was developed based on the Wolf Motor Test to assess the patient’s functional abilities during rehabilitation exercises following clinical procedures [87]. Robotic assistance was adjusted according to the patient’s FAI score, which is determined by parameters such as position error or time to complete the exercise. The method was tested with three hemiplegic patients and during the experiments, the patient’s arm movements were measured using two IMU sensors. The experimental results have shown that the FAI algorithm evaluates patients’ disability levels consistently with clinical methods and the controller adjusts the robotic assistance according to the patient’s FAI score. In another study with patients, Pehlivan et al. improved the mAAN strategy presented in their previous work [39], using a nonlinear disturbance observer (NDO) instead of a Kalman filter to predict patient inputs [88]. NDO has been preferred due to its ability to achieve low prediction errors even in the presence of time-varying disturbances. The method was tested with a SCI patient during wrist flexion–extension exercises. Additionally, during the experiments, the patient’s EMG signals were measured to evaluate muscle activations and patient participation. The results have shown that experiments incorporating the mAAN algorithm led to increased patient participation as determined by EMG signals.

**Table 3:** Pilot clinical trials

Study	Clinical trial group	PPA method	#Patients	Robotic device
Leconte and Ronsse [85]	Pilot study	Performance indicator–based	2	REA-plan end-effector robot
Mounis et al. [87]	Pilot study	Performance indicator–based	3	2 DOF exoskeleton robot
Pehlivan et al. [88]	Pilot study	Force estimation	1	RiceWrist-S
Chia et al. [86]	Pilot study	Force estimation	1	NTUH-II exoskeleton robot
Guidali et al. [89]	Pilot study	Performance indicator–based	2	ARMIN-III
Marini et al. [90]	Pilot study	Position error–based	1	Wrist rehabilitation robotic device

Guidali et al. [89] presented an adaptive patient-cooperative method to support the rehabilitation exercises of ADL for patients. In this approach, patient impairments were estimated and continuously updated using kinematic measurements such as velocity and exercise completion time during exercises, and assistive force was provided based on the patient’s abilities. Additionally, a forgetting factor was included in the method to reduce the assistive force after each patient ability estimation, in line with the AAN strategy, to increase patient participation. The method was tested using the 7-DOF ARMIN III rehabilitation robot with ten healthy participants and two chronic stroke patients. The findings demonstrated that the developed method could estimate the necessary arm support for various ADL movements and provide assistance in accordance with the AAN approach. Marini et al. [90] proposed an AAN strategy in which the assistance provided to the patient is continuously adjusted throughout the rehabilitation trial to increase active participation. The effectiveness of this method was tested in clinical trials with a 14-year-old stroke patient over 3 months using a 3-DOF wrist rehabilitation device. The findings demonstrated that the developed method improved motor coordination and function and reduced spasticity in pediatric stroke patients.

## Challenges and future prospects

AAN strategies are an increasingly widespread approach in robotic rehabilitation [91, 92]. However, as detailed in previous sections, some issues with the current PPA methods implemented in AAN strategies restrict this approach’s future potential. High-tech robotic rehabilitation devices, composed of advanced sensors, controllers, and actuators, are often too expensive for many healthcare institutions, which limits their accessibility. Also, the effectiveness of many methods has not been clinically tested. Of the 51 methods evaluated in this study, only 15 have been clinically tested, accounting for less than 30%. Among the clinically tested methods, position error–based approaches represent the majority, with five different approaches. This prevalence may be attributed to the ease of implementation associated with error-based approaches, which are often simpler to apply in clinical settings. Furthermore, an examination of the clinical studies reveals none of the EMG- or EEG-based approaches have been clinically tested. This can be explained by the challenges posed by EMG and EEG signal measurement techniques, which are often less practical for clinical environments due to their complexity and setup requirements. Parallel group–controlled trials are the most effective approach to evaluating the efficacy of proposed AAN strategies, particularly in comparison to conventional therapy. However, the availability of only four clinical studies using this approach significantly limits the ability to assess the methods’ effectiveness comprehensively. On the other hand, findings from three out of the four performed parallel group–controlled trials indicate that patients who get robot-assisted rehabilitation demonstrated superior outcomes compared to those receiving traditional therapy methods. However, more approaches should be tested clinically, and the long-term advantages and effects of AAN-based robotic rehabilitation approaches over conventional rehabilitation need to be established.

In addition to the lack of clinically tested methods, the classified PPA methods also exhibit certain technical limitations. For instance, in the case of EMG-EEG-based approaches, while EMG signals are generally preferred over EEG signals, as previously mentioned, due to their higher robustness and signal-to-noise ratio, the use of wired sensor systems can be restrictive for patients, particularly during exercises that involve multiple muscle groups [93]. Regarding physiological signals, especially GSR, the sensor placement should be precisely chosen as the signal can be influenced by factors such as sweating and the thickness of the outer skin layer [70]. Also, pulse signals can be affected by movement artifacts during exercises [94]. Additionally, in performance indicator–based approaches, the developed indicators may be difficult or impossible to adapt to different exercises, which restricts the applicability of this approach to various types of therapy.

The applicability of PPA methods can also vary significantly across different patient groups. For example, while EMG signals can be effectively used in the rehabilitation of patients with conditions such as stroke, SCI, and degenerative diseases [95], their use is limited for fine motor movements such as finger and wrist actions [46]. For patients with upper extremity circulation issues, pacemaker users, or pregnant individuals, using physiological responses such as HR and GSR signals for accurate assessment can be challenging. In these populations, the typical physiological patterns may be altered due to underlying conditions, which can interfere with the reliability of measurements. Also, force-based performance assessment can be difficult for certain patient populations. For instance in patients with spastic muscles, increased stiffness, and hyperactive stretch reflexes are often observed, making it challenging to accurately measure the force [96]. Additionally, patients with cognitive impairments may have difficulty understanding or consistently following instructions, which can directly affect the accuracy of force-based assessments [97, 98]. Incomplete SCI (iSCI) patients may also not be suitable for force-based performance assessments due to common symptoms such as muscle weakness and spasticity, which significantly affect the consistency and reliability of force measurements [99].

Different patient populations have varying therapeutic needs. To meet the therapy needs of patients with different motor impairments, more complex devices with higher DOF are being developed [100, 101], which leads to increased computational complexity and more intricate robot dynamics [101]. Also, the increased complexity presents substantial challenges in both the design and control of the robotic devices [102]. To address that issue, artificial intelligence (AI) applications, which are expected to approach human intelligence capacity by the 2040s [103], possess great potential [104, 105]. Specifically, the computational burden arising from complex robot dynamics can overcome using artificial neural networks (ANN) architectures. This could also provide a solution to the need for precise knowledge of robot dynamics in force-based PPA methods. AI-driven systems including deep neural networks (DNNs) can assess movement quality during rehabilitation exercises, classify movements, detect abnormalities, and provide instant feedback to ensure accurate execution of exercises [106]. Neural networks can process signals like EMG and EEG in real time, providing valuable insights into neuromuscular activity, which enables precise adjustments to robotic assistance levels, dynamically tailoring support to meet the patient’s needs [106, 107]. Also, deep learning models can analyze large amounts of rehabilitation data to predict patient performance and recovery trends. Adaptive algorithms, including reinforcement learning (RL) and supervised learning, can help customize therapy plans based on real-time patient feedback [107]. Additionally, AI-based approaches can enable more personalized rehabilitation applications in AAN strategies by adapting assistance not only based on performance but also according to predictive models of the patient’s progress. It will also allow for the dynamic adaptation of therapy task difficulties, further enhancing patient engagement and improving outcomes obtained from therapy.

Future studies should also focus on performance evaluation methods that incorporate approaches considering multiple patient data simultaneously. The multimodal integration of different data, such as EMG signals, physiological responses, and IMU sensors, can help overcome the disadvantages arising from using these data individually. Additionally, evaluating these data together not only allows for a more comprehensive performance assessment but also enables the examination of other factors influencing active participation and performance, such as fatigue and motivation [108–111]. With such multimodal sensor integration approaches, more effective AAN strategies can be developed to address a broader patient population.

Wearable technologies and soft robotic applications are also expected to play a significant role in future applications [112, 113]. Wearable technologies have a rapidly growing market value, with the market size expected to reach US$5.2 billion by 2025 [114]. Devices developed through soft robotic applications will facilitate patient access to therapeutic devices due to their more flexible and cost-effective structures compared to rigid designs. Also, the swiftly evolving wearable technologies provide convenience for home rehabilitation applications, allowing treatment to continue outside clinical settings. This could provide a solution to the increasing demand for remote health care, particularly following the COVID-19 pandemic [115]. The increase in home rehabilitation applications will enable patient monitoring for clinicians and allow the remote management of the therapy process. Additionally, wearable technologies and soft robotic applications provide opportunities for telemedicine and tele-rehabilitation, facilitating the monitoring and evaluation of homecare patients or individuals who face challenges in visiting healthcare facilities. In particular, wireless wearable technologies offer a solution to wired data collection systems, which are one of the limiting factors in EMG and EEG signal measurement. Wearable technologies also enable easier implementation and remote data collection and evaluation within PPA methods based on physiological signals [116, 117].

Active patient participation in exercises is one of the key factors in enhancing the outcomes achieved from therapy. Besides, there are other factors that contribute to improving the functional outcomes of therapy including the exercises being sufficiently challenging for the patient, their relevance to ADL, and the patient’s motivation [118]. In some AAN applications, not only is robotic assistance adjusted based on patient performance, but the difficulty of the therapy tasks is also modified [91, 119]. This approach is beneficial as it ensures the exercises remain challenging for the patient, which is substantial for effective rehabilitation. Additionally, patient motivation during exercises is another crucial factor that should be considered in AAN approaches. Visual and auditory feedback provided to patients about their performance during exercises can positively impact both their engagement and performance [120, 121]. By offering real-time feedback, patients can better understand their progress, make necessary adjustments, and stay motivated throughout the rehabilitation process. Also, selecting therapy exercises that are related to ADL is not only a factor that can contribute to improved outcomes from therapy but will also enhance patient motivation [122, 123].

## Conclusions

This paper has reviewed and classified the PPA methods used in AAN therapy strategies for upper extremity rehabilitation. Although several review studies have been conducted on upper extremity robotic rehabilitation devices, this study is the first to comprehensively and extensively analyze PPA methods used in AAN strategies in such detail. In this study, clinically tested methods were also classified and analyzed, addressing the limitations of current PPA approaches and providing recommendations for improvement. The classification of PPA methods was based on their implementation approaches, including position error–based methods, force-based methods, EMG- and EEG-based methods, performance indicator–based methods, and physiological signal–based methods. Each method has been evaluated for its advantages and shortcomings. Position error–based methods, although widely used, may not provide a complete picture of a patient’s motor skills, as they rely primarily on the difference between the desired and actual movement paths. Similarly, force-based methods offer valuable insight into the physical capabilities of patients but are often limited by the complexity of robotic dynamics. EMG- and EEG-based methods, while promising in capturing muscle activity and brain signals, face challenges related to signal quality and measurement constraints. Performance indicator–based approaches provide an alternative by using multiple indicators to assess therapy performance but may need adjustment depending on the type of exercise. Lastly, physiological signal-based methods offer a unique perspective by measuring emotional and physical responses, though they are susceptible to environmental factors.

Despite significant advancements in AAN strategies, some challenges remain. Many methods have yet to be clinically tested, and their long-term efficacy in comparison to conventional therapies is not fully established. High-tech robotic rehabilitation devices are often expensive, making them inaccessible to many healthcare facilities. Additionally, most PPA methods are tailored to specific exercises or device designs, limiting their applicability across various patient populations. More clinical trials are needed to validate these approaches and expand their generalizability. There is also a need for multimodal data integration, combining different signals such as EMG, EEG, and physiological responses, to provide more comprehensive assessments of patient performance. Moreover, leveraging AI could help reduce the computational complexity of robotic systems, enabling more personalized and adaptive therapy strategies.

In conclusion, while AAN-based robotic rehabilitation presents promising opportunities for enhancing patient engagement and recovery, further clinical validation and innovation in PPA methods are necessary to fully realize its potential. By addressing current limitations, future developments in this field could significantly improve the effectiveness and accessibility of robotic rehabilitation technologies.

## Nomenclature

AAN: Assist-as-needed
ADL: Activities of daily living
AFO: Adaptive frequency oscillator
AI: Artificial intelligence
ANN: Artificial neural network
ARAT: Action research arm test
CPG: Central pattern generation
DNNs: Deep neural networks
DOF: Degrees of freedom
EMG: Electromyography
EEG: Electroencephalography
FAI: Functional ability index
FASF: Functional activity spline function
FIS: Fuzzy inference system
FMA-UE: Fugl-Meyer assessment
gAAN: Greedy AAN
GSR: Galvanic skin response
HR: Heart rate
IEEE: Institute of Electrical & Electronics Engineers
iSCI: Incomplete spinal cord injury
LOC: Level of capability
mAAN: Minimal AAN
M-IMU: Magneto-intertial unit
MLP: Multilayer perceptron
NDO: Nonlinear disturbance observer
NN: Neural network
pAAN: Progress-based AAN
PPA: Patient performance assessment
RBF: Radial basis function
RBFNN: Radial basis function neural network
RIR: Restriction interaction region
RL: Reinforcement learning
ROM: Range of motion
RR: Respiratory rate
PID: Proportional integral derivative
SCI: Spinal cord injury
sEMG: Surface EMG
ST: Subjected-triggered
STREAM: : Stroke rehabilitation assessment of movement
VAS: Visual analog scale
WoS: Web of Science
ZIF: Zero interaction force
